# Multi-Locus Analysis Reveals A Different Pattern of Genetic Diversity for Mitochondrial and Nuclear DNA between Wild and Domestic Pigs in East Asia

**DOI:** 10.1371/journal.pone.0026416

**Published:** 2011-10-31

**Authors:** Yin-Qiu Ji, Dong-Dong Wu, Gui-Sheng Wu, Guo-Dong Wang, Ya-Ping Zhang

**Affiliations:** 1 State Key Laboratory of Genetic Resources and Evolution, Kunming Institute of Zoology, Chinese Academy of Sciences, Kunming, China; 2 The Graduate School of the Chinese Academy of Sciences, Beijing, China; 3 Laboratory for Conservation and Utilization of Bio-resource, Yunnan University, Kunming, China; Tulane University Health Sciences Center, United States of America

## Abstract

**Background:**

A major reduction of genetic diversity in mtDNA occurred during the domestication of East Asian pigs. However, the extent to which genetic diversity has been lost in the nuclear genome is uncertain. To reveal levels and patterns of nucleotide diversity and to elucidate the genetic relationships and demographic history of domestic pigs and their ancestors, wild boars, we investigated 14 nuclear markers (including 8 functional genes, 2 pseudogenes and 4 intergenic regions) from 11 different chromosomes in East Asia-wide samples and pooled them with previously obtained mtDNA data for a combined analysis.

**Principal Findings:**

The results indicated that domestic pigs and wild boars possess comparable levels of nucleotide diversity across the nuclear genome, which is inconsistent with patterns that have been found in mitochondrial genome.

**Conclusions:**

This incongruence between the mtDNA and nuclear genomes is suggestive of a large-scale backcross between male wild boars and female domestic pigs in East Asia. Our data reveal the impacts of founder effects and backcross on the pig genome and help us better understand the complex demographic histories of East Asian pigs, which will be useful for future work on artificial selection.

## Introduction

Domestication has been generally seen as a process that starts with a small number of wild individuals, which through succeeding generations of breeding, are integrated into human societies and thereby lose almost all opportunities to mate with their wild relatives [1], [2], [3], [4], [5]. In view of this, the general expectation is that founder effects should cause a general loss of genetic diversity in domesticated species relative to their wild progenitors due to a domestication bottleneck, and this has been observed in crop plants [6], [7], [8]. However, the impacts of domestication on the genetic diversity of domesticated animals, livestock, is largely unknown since most of the putative wild ancestors of livestock are extinct or are being threatened by extinction and are therefore themselves genetically depauperate [9]. One of the few exceptions is the wild boar, the ancestor of domestic pigs. Both wild boars and domestic pigs are widely distributed in the Old World [10], which provides an excellent opportunity to study the impacts of domestication on livestock genetic diversity.

To date, many previous studies have focused on the origin and the distribution histories of domestic pigs [10], [11], [12], [13], [14], [15], [16], [17], and future work will focus more and more on identifying economically important genes under artificial selection. However, existing evidence has implicated artificial selection as another major cause of founder effects, in addition to domestication bottlenecks [7], [18], [19], [20], [21], [22]. The difference between these two factors is that domestication bottlenecks reduce genetic diversity across the entire genome, while artificial selection is expected to reduce diversity only at selected loci, plus linked regions. This latter pattern has been observed, for example, in maize [7], [21]. Therefore, testing for any domestication bottleneck events in the pig genome will aid ongoing efforts to identify economically important loci that have been subject to artificial selection. If there have been genome-wide domestication bottlenecks, merely showing that any particular locus has low diversity will not in itself be sufficient to conclude that selection has acted on that locus.

Some studies have compared genetic diversity between wild boars and domestic pigs using mtDNA and nuclear DNA markers [14], [16], [23], [24], [25], [26], [27], and these studies have found no evidence for loss of genetic diversity, with the exceptions of Wu et al. (2007) and Scandura et al. (2008). These two studies showed that domestic pigs have lower mtDNA diversity than do wild boars in East Asia (Wu et al. 2007) and in Europe (Scandura et al. 2008). On the other hand, data from autosomal microsatellites, mitochondrial and Y-chromosome polymorphisms in Ramirez et al. (2009) and three nuclear functional genes (*FABP4*, *FABP5* and *IGF2*) in the studies of Ojeda et al. [23], [24], [25] showed that genetic diversity is similar in domestic pigs and wild boars. This difference among the studies might have resulted from different sampling ranges: Wu's dataset was only from Asia, Scandura's was from Europe, Ramirez et al.'s and Ojeda et al.'s were from a much larger range, a sample of pigs and wild boars with a worldwide distribution.

Several investigations on the origin of pigs [10], [11], [12], [28] have shown that pigs have been independently domesticated multiple times in multiple sites, resulting in clear phylogeographic structures in both wild boars and domestic pigs, with multiple, highly differentiated mtDNA gene pools, which, if pooled for analysis, would result in an overestimation of mtDNA diversity and the loss of any signal of founder effects. For this reason, Ramirez et al. (2009) might have overestimated mtDNA diversity of pigs and showed a different result from Wu et al. (2007) and Scandura et al. (2008).

The disagreement between the reduced mtDNA diversity found in Wu et al. (2007) and Scandura et al. (2008) and failure to observe reduced diversity in nuclear DNA [16], [23], [25] might also have resulted from differences in size of sampling ranges. The exception is Ojeda et al. (2008) [24], which compared the genetic diversities of *IGF2* across continents and showed no genetic loss.

**Figure 1:**
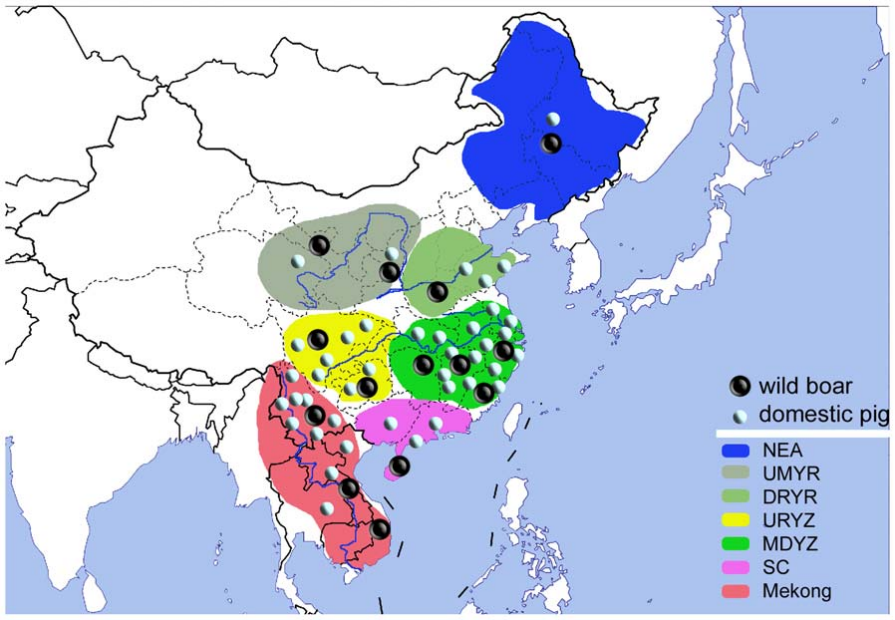
Geographical and group distribution of the East Asian wild boars and domestic pigs sampled. All the samples are sorted into seven groups: NEA = Northeast China, UMYR = the upper and middle catchment of the Yellow River, DRYR = the downstream catchment of the Yellow River, URYZ = the upper catchment of the Yangtze River, MDYZ = the middle and downstream catchment of the Yangtze River, SC = South China, and Mekong = the Mekong River catchment.

It is interesting to find such an unexpected difference between mitochondrial and nuclear DNA, which may suggest different evolutionary histories between male and female lineages during pig domestication. But more data are needed because *IGF2* is a possible selection target locus and cannot represent the whole nuclear genome. Here, we provide a study of the levels and patterns of genetic variation in domestic pigs, from 41 breeds, and wild boars, from 14 localities, in East Asia (Figure 1) that is based on multiple markers. We characterized 14 nuclear markers from 11 different chromosomes, including 8 functional genes, 2 pseudogenes, and 4 intergenic sequences, and pooled them with previously published mtDNA data [14] to compare genetic diversities of domestic pigs and wild boars, to detect any founder effect of pig domestication and to infer any demographic events that have had important impacts on the pig genome.

## Materials and Methods

### Samples and markers studied

In this study, our samples were mostly taken from individuals that were collected from the East Asian mainland and had been used in our previous analysis of mtDNA [14]. Wild boars were from 14 geographic sites and domestic pigs were from 41 local breeds (Figure 1).

**Figure 2:**
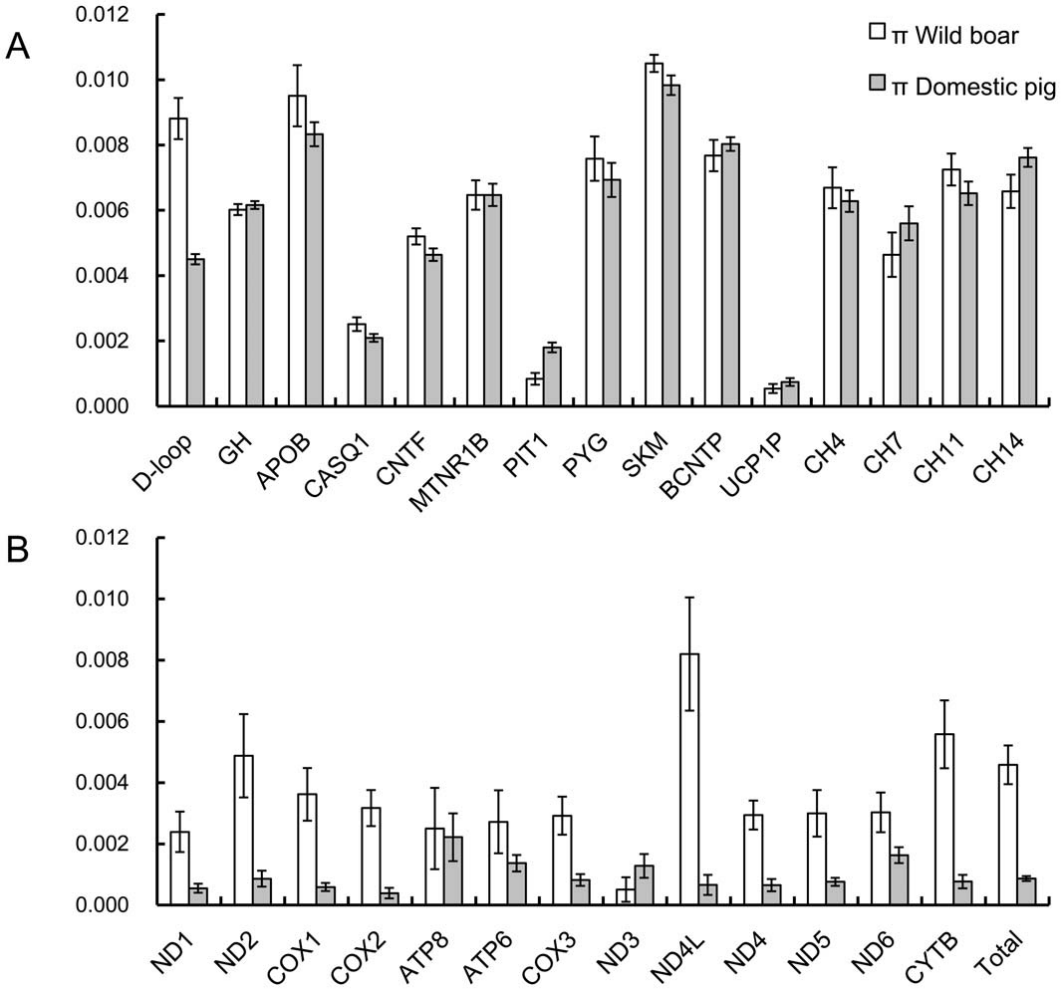
Comparison of π between wild boars and domestic pigs. These comparisons were based on the samples from all East Asia. Error bars indicate one standard deviation of the measurement. (A) Comparison of **π** in *D-loop* and 14 nuclear markers. (B) Comparison of **π** in 13 mitochondrial genes and the whole mitochondrial genome.

Because the genetic diversity of a subdivided population will be overestimated, we first sampled at least five individuals in most geographic populations or breeds for the gene *GH*, a nuclear marker with a similar sample size to that of the D-loop region of mtDNA. The data from both *GH* and *D-loop* showed that wild boars and domestic pigs in East Asia can each be treated as a single population without clear subdivided structures (Figure S1). Therefore, the following analyses were mainly based on the whole East Asian population of wild boars or domestic pigs, and breed information was not used. For other nuclear markers, only 1 to 3 individuals were sampled in most geographic populations or breeds, since this sample size is enough to provide a π value with a relatively low standard deviation (less than 10%) when genetic diversity of the whole population of wild boars or domestic pigs was estimated (Figure 2A). We endeavored to collect samples from unrelated individuals, using information provided by owners and local farmers. Dataset S1 presents detailed information on the samples.

The markers used in this study represent 14 nuclear regions on 11 different chromosomes in the pig. The reference sequences of these nuclear markers were obtained from GenBank [accession numbers: GH - M17704, APOB – M22646, CASQ1 – AJ488283, CNTF – U57644, MTNR1B - AJ276454, PIT1 - U00793, PYG - AJ507153, SKM – U23954, BCNTP – AB213482, UCP1P – DQ372918, CH4 – NW_001886157, CH7 – NW_001886412, CH11 – NW_001885204, CH14 – NW_001885377]. In total, we sequenced 9,859 bp, comprised of 1,872 bp of coding sequence and 7,987 bp of noncoding sequence. Detailed information on the location, functional association [29], [30], [31], [32], [33], [34], [35], [36], [37], length of each marker, and primer sequences used is in Table 1. To ensure that intergenic sequences were not influenced by neighboring functional regions, we chose markers for which there were no genes for at least 10 kb to either side [27]. Genomic DNA was extracted from whole blood, tissue, or hair by standard phenol/chloroform methods [38].

**Table 1:** Summary of the nuclear markers surveyed and the primers used in the study.

Marker	Chromosome Location	Functional Association	Alignment Length (bp)			Primer (5′ to 3′)	Ta
			Total	Coding	Noncoding		
*GH*	12	Growth hormone	1,971	648	1323	pGH1F: GTCGACGGGAACAGGATGAGTGGGAGGAGGTT pGH1R: AAGCTTGCCGGGTCAACCATCATTCAGTGTCT pGH1-2F: CCGAAGATGCTATCAGGTGAGTGTA pGH1-2R: TTGGAGAAGGACAAAGAGGGAAGA pGH1-3F: TGTTTGGCACCTCAGACCGC pGH1-3R: GGGTCAACCATCATTCAGTGTCTA	60°C
*APOB*	3	Apolipoprotein B	645	173	472	APOBF: GCTTGCCGAGTCCTAAC APOBR: GGCCAGTCTGACTATTCTAGTT	52°C
*CASQ1*	4	Calsequestrin 1	583	131	452	CASQ1F: CAAAGCCCAGAGATGTTAAGA CASQ1R: GCCAGATCGGGTTTAGAAT	55°C
*CNTF*	2	Ciliary neurotrophic factor	759	114	645	CNTFF: ATGCCCAGTGGATTTAGTC CNTFR: TGACAGGCCTTAGGTAAAGA	50°C
*MTNR1B*	9	Melatonin receptor 1B	568	320	248	MTNR1BF: CGCAGGAAGGTCAAGTCGGACAA MTNR1BR: GGCGAACGAGGTGAGCGAGAG	60°C
*PIT1*	13	POU-domain protein	650	211	439	PIT1F: CAATACAACATAAACATGAGTAGCGT PIT1R: AGGCTCTGCAAAAGTTACTGATAAGATT	51°C
*PYG*	2	Glycogen phosphorylase	578	101	477	PYGF: AGCCCAGAAATGCGGACA PYGR: GGTAGCCACTCTGCGGTGAT	57°C
*SKM*	1	Skeletal muscle calpain	784	174	610	SKMF: CCCAACACACCAAATAACTAGACA SKMR: GCTTGCCAGAAGTGAATGCTA	56°C
*BCNTP*	1	Bcnt pseudogene for bucentaur	669	0	669	BCNTPF: GGGATGCTATGGAGGAAT BCNTPR: ATGGGAAACAGTCGATGA	51°C
*UCP1P*	8	Uncoupling protein 1 pseudogene	635	0	635	UCP1PF: TTCAGATCCAAGGCAAAT UCP1PR: GTCCACCAAGTTCAGTCAAT	52°C
*CH4*	4	Intergenic sequence	350	0	350	CH4F: TCCGAGCCGTATCTGCAAC CH4R: CAGGGGTTTTCAGGGTTTATG	55°C
*CH7*	7	Intergenic sequence	641	0	641	CH7F: GGGAGGGCAGTCACGAAGTC CH7R: TGTAGAGCAGAAATACACGCAACA	55°C
*CH11*	11	Intergenic sequence	506	0	506	CH11F: TCATGACGGAGTGTACGA CH11R: GCAGCCAATCCAACTTAC	51°C
*CH14*	14	Intergenic sequence	520	0	520	CH14F: GGGCTCTGGGACAGTCTCGT CH14R: TGTCCAGAAGAGGCAAACCCATAA	56°C
Total			9,859	1,872	7,987		

### PCR and sequencing

PCR was performed in a 50 µl reaction mixture containing 50–100 ng genomic DNA, 5 µl 10× buffer, 8 µl 2.5 mMol/L dNTPs, 2 µl of 10 µMol/L each primer, and 2.5 unit of Taq DNA polymerase (TaKaRa Biotech, Dalian, China). Thermocycling was 95°C for 3 min, 35 cycles of 94°C for 30 s, optimal Ta for 30 s (Table 1), and 72°C for 1 to 2 min, and a final extension of 72°C for 5 min. PCR products were purified on spin columns (Watson Biotechnologies, Shanghai) and sequenced on an ABI 3730 automated sequencer (Applied Biosystems), using BigDye v3.1 terminator chemistry (Applied Biosystems). To reduce potential errors from PCR, we independently amplified and sequenced each individual twice.

Direct sequencing of PCR products revealed that many individuals were heterozygous. To determine haplotypes unambiguously, we used two different methods. For the *GH* gene, PCR products of heterozygous samples were cloned into the PMD 18-T Vector (TaKaRa Biotech, Dalian, China) and transformed into *Escherichia coli* JM109 cells (TaKaRa Biotech, Dalian, China). Plasmids were extracted from positive clones and sequenced using the above primers. Typically, 2 to 6 clones were sequenced for each individual to determine the exact point mutations. For all other markers, haplotypes were identified with the software package PHASE v2.1.1 [39] using default options. Only those haplotypes with high probability (P>0.8) were retained for data analyses. All haplotypes have been submitted to GenBank [accession numbers: EU684330–EU684446, GU328959–GU329035 and GU348654–GU348822]. All genotype information for each sample at each marker is presented in Dataset S1. The numbers of sequences obtained for each marker in wild boars and domestic pigs are listed in Table 2.

**Table 2:** Summary statistics for the *D-loop* region and all nuclear markers.

Marker	Wild boars								Domestic pigs								*F_ST_*	*Φ_CT_*
	N	S	π	Θ(MPE)	θ(95%)	*D*	*D* *	*F* *	N	S	π	Θ(MPE)	θ(95%)	*D*	*D* *	*F* *		
*GH*	226	78	6.02	16.7	9.6–25.6	−0.36	0.44	0.09	416	65	6.16	6.8	4.6–10.6	0.6	0.23	0.49	0.88	−0.03
*APOB*	54	25	9.51	10.9	6.5–17.3	0.38	1.46*	1.28	96	21	8.33	10.8	6.6–17.3	0.92	0.48	0.77	2.82	0.90
*CASQ1*	64	9	2.51	3.9	1.8–8.6	−0.62	−0.05	−0.28	108	7	2.09	3.7	1.6–7.0	−0.2	0.3	0.16	6.49	5.74*
*CNTF*	62	12	5.2	4.8	1.9–10.8	1.55	0.91	1.33	106	14	4.64	6.5	4.0–10.9	0.86	0.97	1.11	5.11	3.82
*MTNR1B*	54	11	6.47	5.3	2.4–10.1	1.47	1.44	1.71 *	96	12	6.47	8.1	4.6–13.8	1.5	1.47	1.76*	1.40	−1.14
*PIT1*	58	4	0.84	1.4	0.3–3.4	−0.81	−0.14	−0.4	112	7	1.8	3	1.2–5.7	−0.27	1.18	0.82	4.85	1.24
*PYG*	68	19	7.58	12.5	6.5–18.6	0.32	1.27	1.1	102	18	6.93	5.2	3.0–9.2	0.44	1.2	1.1	0.41	−1.20
*SKM*	62	20	10.5	4.3	2.6–7.8	2.82**	0.92	1.90*	108	20	9.83	3.6	2.3–6.6	2.89**	0.38	1.59	−0.56	−1.34
*BCNTP*	58	14	7.68	3.5	1.6–6.0	2.07*	1.55*	2.04**	112	12	8.03	2.7	1.2–5.3	3.58***	1.46	2.65**	2.79	1.44
*UCP1P*	56	4	0.54	1.7	0.4–3.9	−1.34	−0.13	−0.59	94	4	0.74	1.7	0.6–3.7	−0.81	−0.25	−0.5	1.52	−0.98
*CH4*	56	11	6.69	9.9	3.7–19.1	−0.06	−0.33	−0.29	202	12	6.28	9.6	3.7–17.4	0.19	1.43	1.16	6.45	5.07
*CH7*	56	15	4.64	6.5	3.2–12.1	−0.27	0.63	0.38	104	19	5.6	9.5	5.0–14.9	−0.06	0.78	0.56	0.52	−3.13
*CH11*	60	21	7.25	10.7	4.6–21.1	−0.58	−0.16	−0.37	204	17	6.52	10.9	5.1–17.3	0.37	−0.06	0.13	2.30	1.12
*CH14*	54	12	6.58	6.4	4.0–11.5	0.87	1.48	1.51	96	15	7.62	10.7	6.9–19.9	0.99	0.51	0.82	1.00	−0.62
*D-loop*	106	34	8.81	26.2	21.9–41.4	−0.99	−1.69	−1.67	197	21	4.5	14.4	11.6–19.9	−0.52	0.79	0.33	6.90	6.19***

N, number of sequences; S, number of SNPs (excluding insertion-deletions); π, nucleotide diversity estimated in DnaSP; θ, most probable estimate in LAMARC; θ(95%), 95% credible intervals; all π and θ are ×10^3^; D, Tajima's D-statistic; D*, Fu and Li's D-statistic; F*, Fu and Li's F-statistic; F_ST_ and Φ_CT_, genetic differentiation between wild boars and domestic pigs; all F_ST_ and Φ_CT_ are ×10^2^;

*, *P*<0.05;

**, *P*<0.01;

***, *P*<0.001.

### Data analysis

The mtDNA data of Wu et al. (2007) were reanalyzed here because the emphasis before was placed on the origin of domestication. Sequence data from the 14 nuclear markers were assembled and edited using seqman (DNASTAR, Madison, WI), and were aligned using a combination of methods implemented in ClustalW 1.81 [40] and BioEdit version 5.0.9 [41], with further manual refinements. The alignments are available from the authors upon request.

For the analyses of population structure, we placed our samples into the following groups (Figure 1) according to principles previously described in [14] (geographic fauna and possible pig domestication sites): (1) Region NEA, Northeast China, including Jilin, Liaoning, Heilongjiang, northeast Inner Mongolia; (2) Region UMYR, the upper and middle catchment of the Yellow River, including Gansu, east Qinghai, northwest Sichuan, south Inner Mongolia, Ningxia, Shaanxi, Shanxi, and west Henan; (3) Region DRYR, the downstream catchment of the Yellow River, including east Henan, Hebei, and Shandong; (4) Region URYZ, the upstream catchment of the Yangtze River, including Sichuan, Chongqing, Guizhou, northeast Yunnan, west Hubei, and northwest Hunan; (5) Region MDYZ, the middle and downstream catchment of the Yangtze River, including east Hubei, northeast Hunan, Anhui, Jiangxi, Fujian, Zhejiang, Jiangsu, and Shanghai; (6) Region SC, South China, including Guangdong, south and southeast Guangxi, south Hunan and Hainan; (7) Region Mekong, including northwest, south and southeast Yunnan, Laos, Vietnam and Thailand.

We used Bayesian clustering within STRUCTURE version 2.3.3 [42] to deduce if subpopulations exist in East Asian samples. The D-loop region was used to detect mtDNA subdivision and the four intergenic regions combined were used to detect nuclear DNA subdivision. The analyses were performed by using the data of boar-only, pig-only and all samples combined. Structure clusters based on a user-supplied number of populations K and are given LnP(X/K), the log-likelihood of the posterior probability for each K, which is used to discern the optimal number of population clusters. We ran 1,000,000 steps after a burn-in of 100,000 under the admixture model without population data. We ran 20 separate iterations for each value of K from 1 to 20 and used the average value of LnP(X|K) to select K in addition to ΔK [43].

In addition, a median-joining network [44] was drawn for each nuclear marker using the program Network 4.5 to visualize phylogeographic structure within East Asia and the relationship between wild boars and domestic pigs (Figure S1).

To apportion the variation between groups (defined above), within and between populations (breeds in domestic pigs and geographic subpopulations in wild boars), and to estimate genetic differentiation (Φ_CT_) between wild boars and domestic pigs, analyses of molecular variance (AMOVA) [45] were carried out in ARLEQUIN version 3.01 [46] (Dataset S2).

For 14 nuclear markers and the mtDNA D-loop region, we calculated the number of segregating sites, the number of haplotypes, and nucleotide diversity, π (the average number of nucleotide substitutions per site between two sequences) [47]. The level of LD (linkage disequilibrium) was estimated by determining the significance of the associations between all the possible informative nucleotide position pairs within markers with Fisher's exact test after Bonferroni correction. In each marker, the tests of Tajima's *D* [48] and D* and F* of Fu and Li [49] were performed in wild boars and domestic pigs separately. We also calculated the estimator of genetic differentiation (*F_ST_*) at each marker between wild and domestic pigs. For 8 functional genes, N_A_ (the number of nonsynonymous mutations), N_S_ (the number of synonymous mutations) and the ratio of N_A_/N_S_ were estimated. All the above analyses were carried out in DnaSP 5.00 [50].

To investigate the genetic pattern of the 13 mitochondrial coding genes, we chose all the East Asian samples that had a complete mitochondrial genome sequence in Wu et al. (2007), including 11 wild boars and 30 domestic pigs. Detailed information on samples can be found in Wu et al. (2007). For these 13 mitochondrial coding genes and the whole mitochondrial genome, we calculated π, θ (the proportion of segregating sites) [51], N_A_ (the number of nonsynonymous mutations), N_S_ (the number of synonymous mutations) and the ratio of N_A_/N_S_. All these analyses were carried out in DnaSP 5.00.

We used Modeltest 3.7 [52] and PAUP 4b10 [53] to identify the best-fit model parameters, which were used to simulate a model in LAMARC 2.1.3 [54], using Bayesian MCMC analyses with 10,000 recorded genealogies sampled every 20 steps with a burn-in of 1,000 genealogies, to estimate the neutral parameters θ (4N_e_μ for autosomal markers and 2N_f_μ for mtDNA, N_f_ = effective population size of females and μ = mutation rate), the migration rates M (m/μ) from wild boars into domestic pigs, and the recombination rates r (ρ/μ). Analyses were repeated three times to verify that parameters estimated converged within and among runs. The migration rate M (m/μ) was multiplied by θ (4N_e_μ/2N_f_μ) of domestic pigs to calculate M_N_ (4N_e_m/2N_f_m, independence from mutation rate), the average number of effective migrants per generation.

We calculated allele frequency of each SNP that we found in the 14 nuclear markers and used a chi-square test to examine the significance of the allele frequency changes in all the SNP sites between wild boars and domestic pigs. In addition, we calculated and compared LD ratios (the proportion of significant pairwise comparisons to total possible pairwise comparisons) between wild boars and domestic pigs.

## Results and Discussion

### Population structure

Before estimating and comparing genetic diversity between wild boars and domestic pigs, we first investigated population structure within East Asia. If the population is subdivided, all diversity analyses should be performed within each subpopulation.

**Figure 3:**
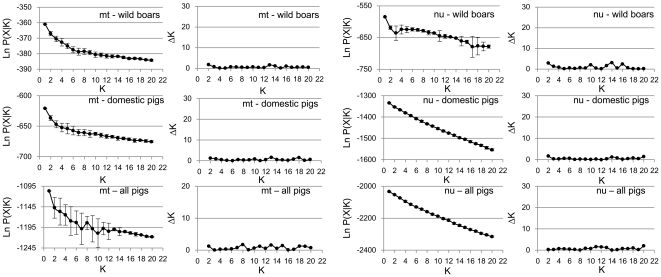
Bayesian clustering results. Plots of LnP(X|K) (±SD) and ΔK versus the number of K at *D-loop* (mt) or in multi locus (*CH4*, *CH7*, *CH11*, and *CH14*) nuclear DNA (nu) for wild boars, domestic pigs and both combined.

To start, we used the clustering method in STRUCTURE [42] to deduce the optimal number of subpopulations (K) in East Asian samples. We conducted the analyses for wild boars and domestic pigs separately in addition to all samples combined since the hierarchical structure of wild boars or domestic pigs might not be detected if they both were combined into a single analysis. Because selection might confound the detection of substructure, only *D-loop* and the four nuclear intergenic markers that are considered to be neutral were used. The results are shown in Figure 3. For all the structure analyses, the K with the highest LnP(X|K) and lowest standard deviation was 1. Although the method of ΔK [43] did not give the corresponding value of K = 1, all the plots of ΔK versus K show no peak from K = 2 to 20, further suggesting that 1 is the correct K number. The results of structure analyses not only indicated no substructure in either wild boars or domestic pigs but also showed a very high level of admixture between boar and pig.

Furthermore, the networks of all these markers do not show a clear phylogeographic structure in either wild boars or domestic pigs of East Asia (Figure S1), which is unlike the strong structure of pig mitochondrial sequences globally [10], probably resulting from high gene flow among different populations in the East Asian mainland.

To further investigate population structure in East Asia, we performed AMOVA analyses on all markers in wild boars and domestic pigs separately. The results are in Dataset S2 and show that for all the markers analyzed, most genetic variation derives from the difference within populations in both wild boars and domestic pigs. In wild boars, only three genes (*APOB*, *CNTF* and *PIT1*) show significant genetic differentiation among groups (P<0.05), whereas in domestic pigs, only *D-loop* does so, which might be the result of subdivision since the D-loop region is usually considered a neutral marker. Overall, most AMOVA results show low genetic differentiation among East Asian groups, which is consistent with the results of STRUCTURE and the network analysis. Consequently, the following analyses were based on the whole sampled population of East Asian wild boars or domestic pigs.

### Comparison of genetic diversity between wild boars and domestic pigs

The loss of genetic diversity due to founder effects is considered to be a general consequence of domestication, so the most straightforward strategy for testing for the effect of domestication is to compare nucleotide diversity between domestic species and their wild progenitors.

For *D-loop* and each nuclear marker, estimated nucleotide diversity (π) is listed in Table 2. For the 13 mtDNA coding genes and the whole mitochondrial genome, π is listed in Table 3. A summary comparison of π values between wild boars and domestic pigs is shown in Figure 2. *D-loop* and most mtDNA coding genes (*ND1*, *ND2*, *COX1*, *COX2*, *COX3*, *ND4L*, *ND4*, *ND5*, *ND6*, and *CYTB*) confirm that domestic pigs have a clearly lower level of genetic diversity than do wild boars, but nuclear DNA data reveal that domestic pigs and wild boars exhibit comparable levels of diversity in all the markers except *PIT1*, which shows higher diversity in domestic pigs than in wild boars.

**Table 3:** Some statistics of all the 13 protein-coding mitochondrial genes and the whole mitochondrial genome.

Marker	Wild boars					Domestic pigs				
	π	θ	N_A_	N_S_	N_A_/N_S_	π	θ	N_A_	N_S_	N_A_/N_S_
*ND1*	2.39	3.21	0	9	0.00	0.55	1.58	1	5	**0.20**
*ND2*	4.88	5.89	6	12	0.50	0.86	1.93	5	3	**1.67**
*COX1*	3.62	4.64	1	20	0.05	0.59	1.8	4	7	**0.57**
*COX2*	3.17	5.95	3	9	0.33	0.39	1.47	1	3	0.33
*ATP8*	2.5	3.35	2	0	NA	2.22	6.19	4	1	4.00
*ATP6*	2.72	4.01	3	5	0.60	1.37	2.59	6	1	**6.00**
*COX3*	2.92	3.92	4	5	0.80	0.82	1.93	1	5	0.20
*ND3*	0.51	0.96	1	0	NA	1.28	3.54	3	2	1.50
*ND4L*	8.2	9.2	2	6	0.33	0.66	1.7	0	2	0.00
*ND4*	2.94	3.65	12	4	3.00	0.65	1.8	8	2	**4.00**
*ND5*	3	4.5	9	15	0.60	0.76	1.8	3	10	0.30
*ND6*	3.03	4.53	6	1	6.00	1.63	1.91	3	1	3.00
*CYTB*	5.58	7.79	5	21	0.24	0.77	2.07	5	4	**1.25**
Total	4.58	4.58	53	105	0.50	0.87	1.98	43	45	**0.96**

π and θ, nucleotide diversity estimated in DnaSP and are ×10^3^; N_A_, the number of nonsynonymous mutations; N_S_, the number of synonymous mutations; NA, not available.

The N_A_/N_S_s that were larger in domestic pigs were highlighted in bold.

**Figure 4:**
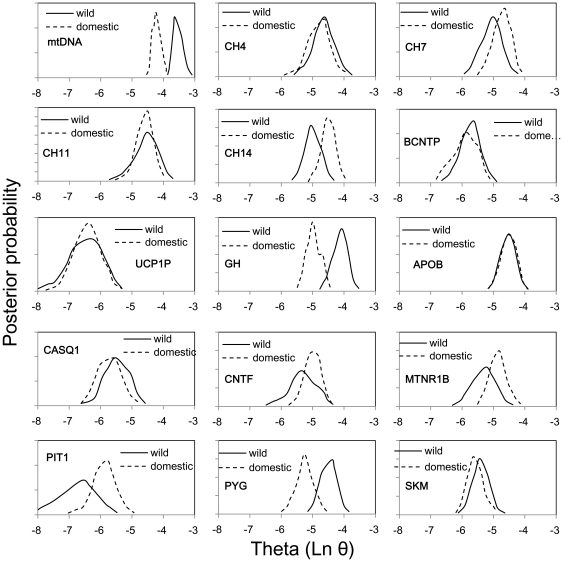
Comparison of θ in *D-loop* and 14 nuclear markers between wild boars and domestic pigs. The values of θ (4N_e_μ for autosomal markers and 2N_f_μ for mtDNA, N_f_ = effective population size of females and μ = mutation rate) were calculated by using Lamarc analysis.

Given that the demographic histories of wild and domestic pigs are quite complex, we also used Bayesian MCMC analysis, which has the advantage of explicitly handling uncertainty in parameter estimates, implemented in LAMARC, to estimate genetic diversities θ at *D-loop* and at each nuclear marker. The θ values are listed in Table 2, and comparisons between wild and domestic pigs are shown in Figure 4. LAMARC analyses show that the θ of *D-loop* in wild boars is higher than that in domestic pigs without overlap in the 95% confidence interval (CI), but overlapping CIs are observed in all the nuclear markers. Based on the most probable estimates (MPEs) of θ, domestic pigs have retained 55% (θ = 0.0144) of the mtDNA diversity present in wild boars (θ = 0.0262) but have a similar or slightly higher diversity than wild boars for most nuclear markers, which is consistent with the data from π (Figure 2A).

Overall, the mtDNA data confirm previous results [14] in providing strong evidence for the loss of mtDNA genetic diversity in domestic pigs, which could result from the founder effects of domestication bottlenecks and/or from artificial selection. However, in the nuclear genome, regardless of whether we analyzed intergenic markers, pseudogenes, or functional genes, all but one (*PIT1*) reveal that domestic pigs and wild boars have indistinguishable levels of nuclear genetic diversity.

### Founder effects of domestication in nuclear DNA

Even though reduced genetic diversity was not detected in nuclear DNA, data from low-frequency alleles nonetheless supports the persistence of at least some founder effects. Our analyses found that the 131 SNP alleles (excluding indels) with frequencies <10% were detected across all the nuclear markers in wild boars, but only 83 were present in domestic pigs (Dataset S3), a 36.6% loss of low-frequency alleles in domestic pigs. Meanwhile, Tajima's D appears to be higher in domestic pigs than in wild boars for most markers (Table 2), which is also consistent with the loss of low-frequency alleles. Out of all 293 SNPs (excluding indels), 102 (34.8%) had significantly different frequency distributions across pig and boar (Dataset S3). In addition, 10 of 14 nuclear markers in domestic pigs were detected to have a higher proportion of pairs with significant LD than in wild boars (Table 4), suggesting a slight trend toward increasing LD in nuclear DNA during domestication or breeding, which is consistent with founder effects.

**Table 4:** Linkage disequilibrium.

Marker	Wild boars			Domestic pigs		
	P_S_ a	P_T_ b	ratio^c^	P_S_ a	P_T_ b	ratio^c^
*GH*	220	2926	7.52%	439	2080	**21.11%**
*APOB*	47	300	15.67%	49	210	**23.33%**
*CASQ1*	1	36	2.78%	1	21	**4.76%**
*CNTF*	15	66	22.73%	14	91	15.38%
*MTNR1B*	11	55	20%	16	66	**24.24%**
*PIT1*	1	6	16.67%	2	21	9.52%
*PYG*	31	171	18.13%	37	153	**24.18%**
*SKM*	119	190	62.63%	119	190	62.63%
*BCNTP*	51	91	56.04%	66	66	**100%**
*UCP1P*	0	6	0%	0	6	0%
*CH4*	3	55	5.45%	16	66	**24.24%**
*CH7*	21	105	20%	38	171	**22.22%**
*CH11*	8	210	3.81%	42	136	**10.88%**
*CH14*	12	66	18.18%	22	105	**20.95%**
*D-loop*	49	528	9.28%	9	210	4.29%

a Ps, number of significant pairwise comparisons by Fisher's exact test after Bonferroni correction.

b P_T_, number of all possible pairwise comparisons.

c ratio = Ps/P_T_.

The ratios that were greater in domestic pigs were highlighted in bold.

### What caused the incongruence between mtDNA and nuclear DNA?

If only the nuclear data are considered, the observation of no loss in overall genetic diversity in domestic pigs relative to wild boars could be explained by post-domestication bottleneck events *in wild boars*, such as widespread hunting. It is plausible that such bottlenecks have occurred in East Asian populations of wild boars, but such bottlenecks cannot explain the inconsistency between mtDNA and nuclear DNA. A bottleneck event will affect mitochondrial and nuclear genomes simultaneously and should in fact affect the mitochondrial genome more strongly because of its smaller effective population size. Thus, what has caused the incongruence between mtDNA and nuclear DNA?

**Figure 5:**
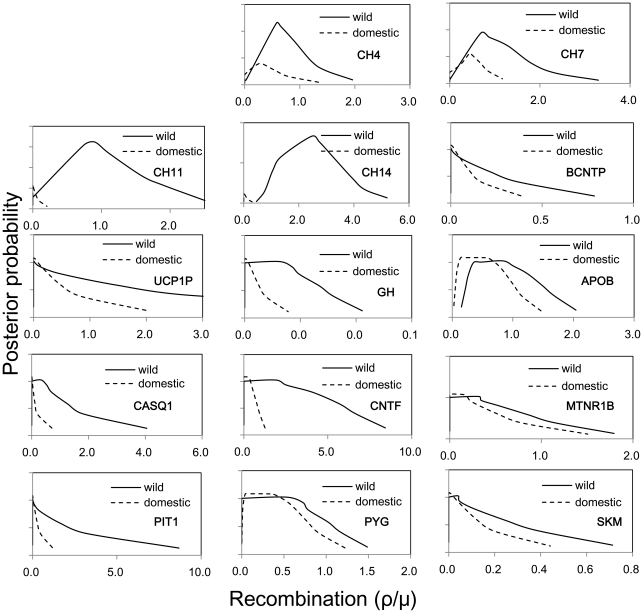
LAMARC analysis showing the comparison of recombination estimates between wild and domestic pigs. Recombination rates were calculated and compared at all the 14 nuclear markers.

There are three major factors influencing genetic diversity: recombination, selection and demographic events. Firstly, to detect if recombination has increased nuclear genetic diversity in domestic pigs, we calculated the recombination rates (r) of each nuclear marker by using Bayesian MCMC analysis implemented in LAMARC and compared r between wild boars and domestic pigs. We did not calculate the r of *D-loop* because mtDNA is almost entirely inherited from one parent only. The results showed that 95% CIs of recombination rates overlap between wild and domestic pigs for all comparisons (Figure 5), indicating no elevated recombination rate in the nuclear genome of domestic pigs relative to that of wild boars. This suggests that recombination is not a likely explanation for the similar genetic diversity between pigs and boars.

Selection could also potentially explain the incongruence between mitochondrial and nuclear DNA in two ways. Since mtDNA is inherited without recombination, selection at protein-coding mtDNA loci may reduce genetic diversity across the entire mtDNA genome. However, the samples sequenced for the whole mitochondrial genome were chosen based on the data of the D-loop region to avoid the same haplotype between individuals, which might affect the results of statistical methods that capture the information about the frequency spectrum of alleles. Because of this, we only performed the tests of Tajima's *D* [48] and D* and F* of Fu and Li [49] for *D-loop*, and we calculated and compared the ratios of N_A_/N_S_ at each mitochondrial gene and the whole mitochondrial genome. The results of tests (Table 2) showed no significant departures from neutral expectation in either wild or domestic pigs. From this, we could not determine if mtDNA was under diversity-reducing selection because signatures of selection are always confounded by the effects of historical demographic factors. Domestication is a complicated process including many demographic factors, which could blur the signal of selection. However, comparisons of the N_A_/N_S_ ratio, which is robust to demographic influences, showed that domestic pigs have a higher ratio than wild boars in the total genome and in over half of comparable genes (6/11 = 54.55%) (Table 3), reflecting a general relaxation of selection on the mitochondrial genome of domestic pigs, similar to that observed in dog domestication [55]. By itself, reduced selective pressure can result in higher genetic diversity. In addition, the low value of LD in domestic pigs (Table 4) excludes the hypothesis of a selective sweep in the D-loop region. Thus, on balance, the available data do not suggest that selection has led to reduced mtDNA diversity in domestic pigs, and if anything, reduced selective pressure has resulted in the opposite pattern.

Another hypothesis is that artificial selection has increased the genetic diversity of domestic pigs at the nuclear markers used in this study. First, we performed the tests of Tajima's *D* [48] and Fu and Li's D* and F* [49] for each nuclear marker, and we found that most markers do not show significant departures from neutral expectations in either wild boars or domestic pigs (Table 2). The four exceptions are three functional genes (*APOB*, *MTNR1B* and *SKM*) and one pseudogene (*BCNTP*). Two of these loci, *SKM* and *BCNTP*, are located on the same chromosome (Table 1) and present similar statistics (Table 2). To elucidate the relationship between *SKM* and *BCNTP*, we conducted a NCBI BLAST search in the pig genome database and found that both markers are located in the same genomic contig [GenBank: NW_001885768], indicating that these two markers are closely linked and can be influenced together. Although these four markers were found to have significant departures from neutrality, their diversity was not necessarily influenced by artificial selection. At *APOB*, only wild boars were found to have significant departure, and at the other three loci, both domestic pigs and wild boars show similar departures from neutrality. We then calculated and compared the ratios of N_A_/N_S_ at the 8 functional nuclear genes. The results are different from mtDNA in that domestic pigs had a *lower* level of N_A_/N_S_ than did wild boars in the only 2 genes that showed difference in ratio between wild and domestic pigs (Table 5). This suggests that there has been no relaxation of selection in nuclear DNA in domestic pigs and that there might even be a history of selection at some nuclear loci, which should decrease domestic pig genetic diversity. In addition, most nuclear markers, including most functional genes, pseudogenes, and all intergenic regions from 11 different chromosomes exhibit the same incongruence with the mitochondrial DNA, strongly suggesting that the entire nuclear genome in domestic pigs exhibits high genetic diversity, which is unlikely to be explained by selection. Furthermore, all the intergenic region markers do not exhibit high levels of LD, suggesting that they are not influenced by selection acting on their neighboring functional regions and are therefore neutral. Thus, we reject the hypothesis of selection increasing genetic diversity in domestic pigs.

**Table 5:** Comparison of ratio N_A_/N_S_ between wild and domestic pigs at nuclear functional genes.

Marker	Wild boars			Domestic pigs		
	N_A_	N_S_	N_A_/N_S_	N_A_	N_S_	N_A_/N_S_
*GH*	9	11	0.82	6	11	0.55
*APOB*	6	3	2	5	3	1.67
*CASQ1*	0	0	NA	0	0	NA
*CNTF*	0	1	0	0	1	0
*MTNR1B*	0	3	0	0	3	0
*PIT1*	0	0	NA	1	0	NA
*PYG*	2	0	NA	2	0	NA
*SKM*	0	0	NA	0	0	NA
*Total*	17	18	0.94	14	18	0.78

NA, not available.

We therefore lean toward accepting the last factor, demographic events, to explain the incongruence between mtDNA and nuclear DNA. Both population subdivision and genetic introgression can, under some conditions, increase total population diversity. If population subdivision is the cause, we would expect DNA regions with higher mutation rates to have a higher level of polymorphisms, due to genetic drift [56]. Given that mtDNA usually evolves faster than nuclear DNA [57], [58] and that the AMOVA analyses showed that mtDNA had a higher degree of subdivision than nuclear DNA in domestic pigs, the estimated mitochondrial diversity of domestic pigs should be higher than that of the nuclear genome. However, the opposite is observed in our data. Therefore, we infer that genetic introgression is the main cause of incongruence between mtDNA and nuclear DNA. Inputs of genetic diversity could either originate from other centers of domestication or from wild boars. We lean toward wild boars because there is no evidence of introgression in East Asian local breeds from other centers based on the microsatellite data [16], [59].

**Figure 6:**
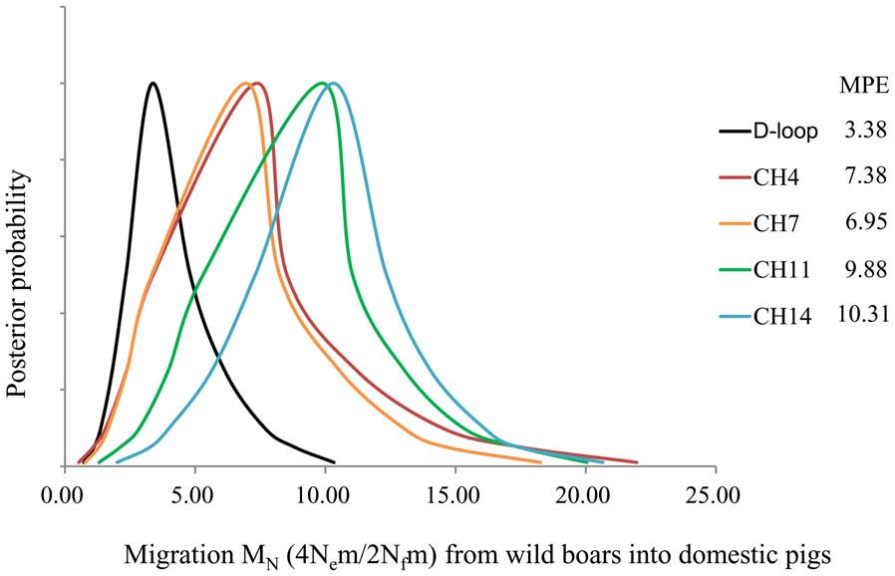
LAMARC analysis showing the migration estimates from wild boars into domestic pigs. The migration estimates were only calculated at 4 intergenic regions and the mtDNA D-loop region.

To further evaluate the hypothesis of genetic introgression from wild boars to domestic pigs, we estimated migration for the mtDNA D-loop region and for four nuclear intergenic regions that are considered to be neutral and therefore free of effects of selection. The results showed that the confidence intervals of M_N_ overlapped for all these regions, but the MPEs of nuclear regions were at least 2 times that of mtDNA (Figure 6), indicating that the number of effective male migrants was similar or higher than that of female migrants per generation. And if independent from effective population size (m = M_N_/4N_e_), the true migration rate of nuclear DNA is probably higher and the male migrants greater because there has been a sex bias (N_f_>N_m_) in domestic pigs in East Asia for thousands of years [48] and the effective population size N_e_ should be smaller than 2N_f_ [49]. Consequently, it is highly possible for males to have contributed more to gene flow from wild into domestic pigs.

### The backcross hypothesis

Backcross is defined here as post-domestication introgression with sex bias. Vila et al. [60] previously proposed that a high level of diversity in nuclear DNA (but not in mtDNA) resulted from mating between wild progenitor males and domesticated females. However, Vila et al.'s analysis was based on MHC, the high diversity of which is maintained by balancing selection. Such markers might therefore overestimate the founder number of domestic pigs because in their simulations, the maximum number of founder populations tested was six, which might underestimate the real number of origins of pig domestication [10], [15], [17]. Our study provides more and stronger evidence in favor of Vila et al.'s proposal of backcross: 1) higher diversity in nuclear DNA relative to mtDNA; 2) shared haplotypes, showing that domestic pig samples from Northeast Asia (NEA), the Yellow River catchment (UMYR and DRYR) and the upstream catchment of the Yangtze River (URYZ) share some haplotypes with the local wild boars in nuclear DNA (Figure S1), but none of them share even the same haplogroup in mtDNA [14]; 3) analyses of migration rates, showing that the male lineage highly possibly has a greater gene flow than female lineage from wild into domestic pigs, which is inconsistent with the observation that the domesticated pig populations typically retain more females than males for production [61], [62]. Furthermore, the hypothesis of backcross is consistent with the observation that pig husbandry in China historically allowed pigs to range freely, and this custom persists in some regions today.

### Conclusions

Although backcrossing has been demonstrated for some domestic species [63], [64], the extent of its impacts to the gene pool of modern livestock was uncertain. The main objective in this study is to investigate its impacts on the domestic pig genome in addition to supporting its existence in pigs. Our data are consistent with the hypothesis that backcross events have increased the diversity of nuclear DNA in East Asian domestic pigs, resulting in a different genetic pattern between male and female lineages. Therefore, any founder effects of a domestication bottleneck have been weakened, resulting in no observed loss of overall nuclear genetic diversity. In addition, the high diversity with a very weak population structure that is observed in nuclear DNA of domestic pigs is expected when most polymorphisms are from wild boars through backcross and not from artificial selection or subdivision in breeding. In conclusion, our data help us better understand the complex demographic histories of East Asian pigs, and bring a practical benefit for the future work on artificial selection. High nuclear genetic diversity increases statistical power in the search for genes that have been subject to artificial selection during domestication, since there is less need to consider the alternative hypothesis of founder effects as a cause of lower diversity in candidate loci for artificial selection.

## Supporting Information

Figure S1 The network of 14 nuclear markers and D-loop in domestic pigs and wild boars of East Asia. These samples are from Northeast Asia (NEA), the upper and middle catchment of the Yellow River (UMYR), the downstream catchment of the Yellow River (DRYR), the upstream catchment of the Yangtze River (URYZ), the middle and downstream catchment of the Yangtze River (MDYZ), the Mekong catchment and South China (SC). Each haplotype is represented by a circle, with the area of the circle proportional to its frequency. Samples from different regions were indicated by different colours. The length of each branch was proportional to the number of mutations on the respective branch. The network of *D-loop* was cited from Wu et al. (2007).
(PDF)

Dataset S1 Sample information. List of the detail information of samples used in this study. The samples of domestic pigs were named by their breeds, following the number code. For each individual, haplotype/genotype is given at each marker. Furthermore, in the mtDNA D-loop region, the clade in Larson et al. (2010) that each individual belongs to is also given.
(XLS)

Dataset S2 AMOVA results. This file listed the AMOVA results of wild boars and domestic pigs. All samples are classified into 7 geographic groups, which have been defined in materials and methods. Populations are divided based on sampling plot or breed. The P-values of Φ_CT_ lower than 0.05 are emphasized by yellow background.
(XLS)

Dataset S3 The allele frequency of every SNP site in wild boar and domestic pig. This file listed and compared the allele frequency of every SNP site between wild boar and domestic pig. The alleles which have a frequency lower than 0.05 in wild boars were highlighted in yellow. The low-frequency alleles which were not present in domestic pig were highlighted in red. χ^2^ test was performed to examine the significance of allele frequency changes in all SNPs, and the changes that were significant were highlighted in purple.
(XLS)
