# Supplementary material for: Multi-Locus Analysis Reveals A Different Pattern of Genetic Diversity for Mitochondrial and Nuclear DNA between Wild and Domestic Pigs in East Asia
Source: PLoS One. 2011 Oct 31;6(10):e26416. doi: 10.1371/journal.pone.0026416 (PMC3204973; doi:10.1371/journal.pone.0026416)
Supplement: Figure S1 — The network of 14 nuclear markers and D-loop in domestic pigs and wild boars of East Asia. These samples are from Northeast Asia (NEA), the upper and middle catchment of the Yellow River (UMYR), the downstream catchment of the Yellow River (DRYR), the upstream catchment of the Yangtze River (URYZ), the middle and downstream catchment of the Yangtze River (MDYZ), the Mekong catchment and South China (SC). Each haplotype is represented by a circle, with the area of the circle proportional to its frequency. Samples from different regions were indicated by different colours. The length of each branch was proportional to the number of mutations on the respective branch. The network of D-loop was cited from Wu et al. (2007). (PDF) [file pone.0026416.s001.pdf]

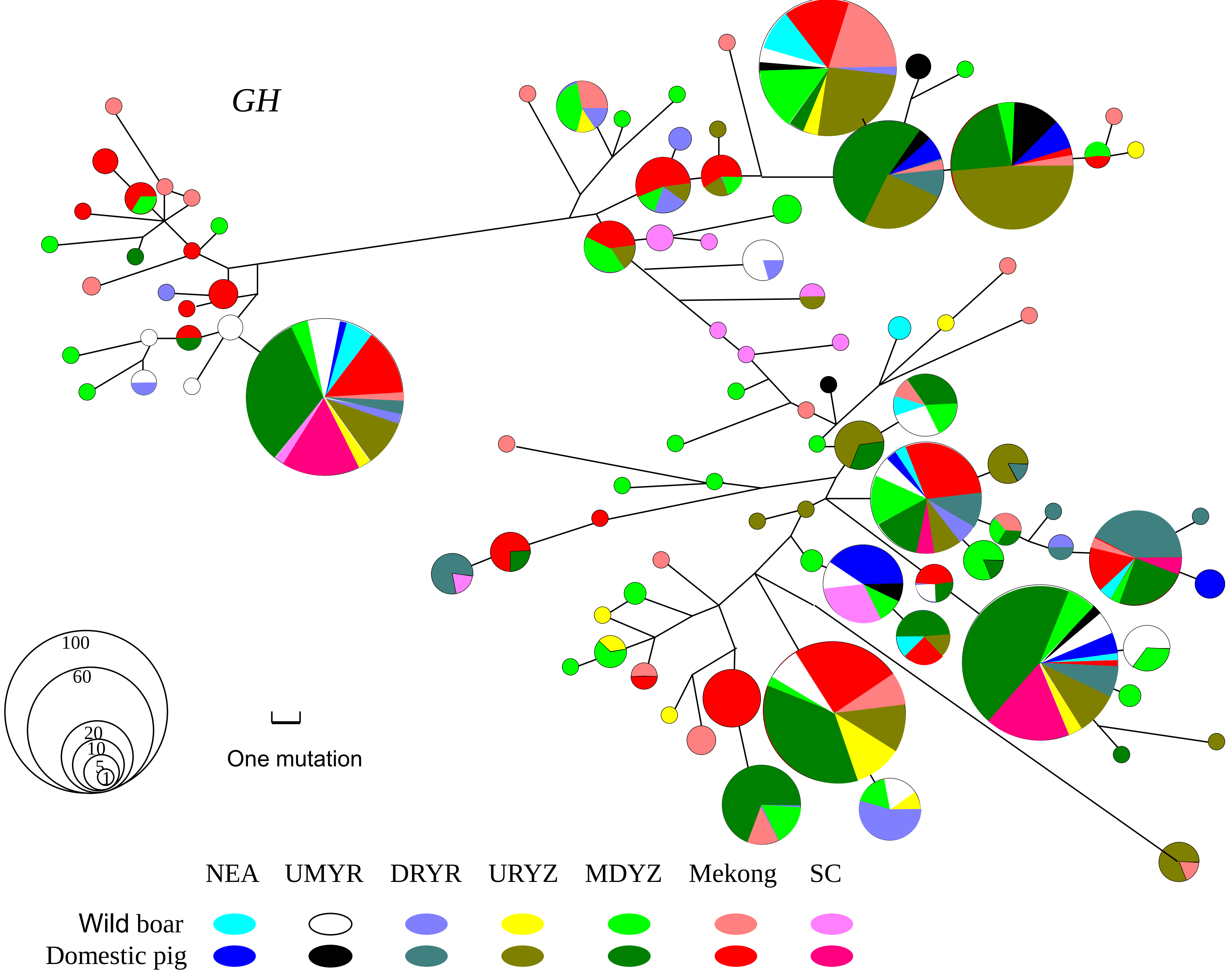

GH
100
60
20
10
5
1
One mutation
NEA
UMYR
DRYR
URYZ
MDYZ
Mekong
SC
Wild boar
Domestic pig

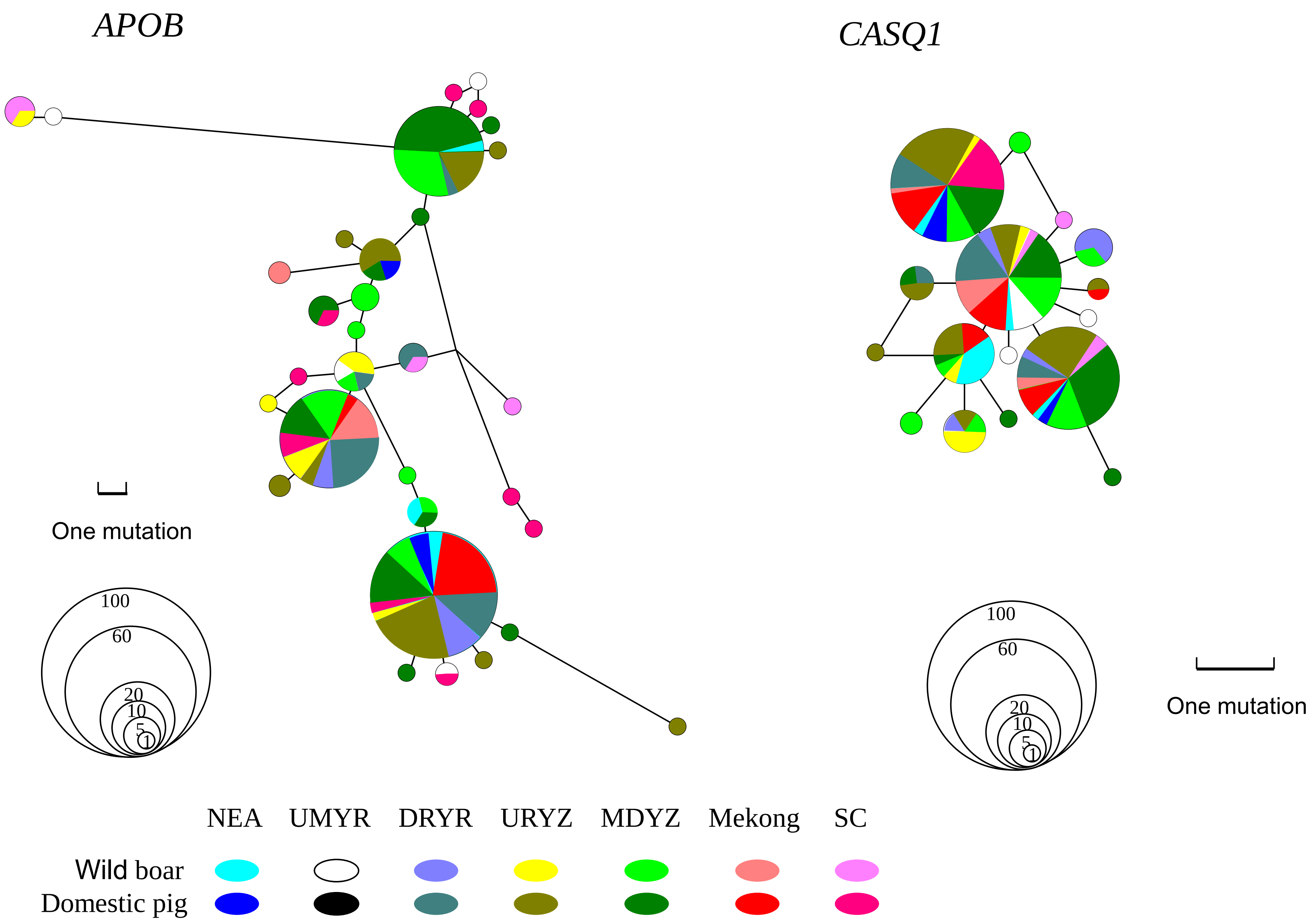

APOB
CASQ1
One mutation
100
60
20
10
5
1
One mutation
100
60
20
10
5
1
NEA
UMYR
DRYR
URYZ
MDYZ
Mekong
SC
Wild boar
Domestic pig

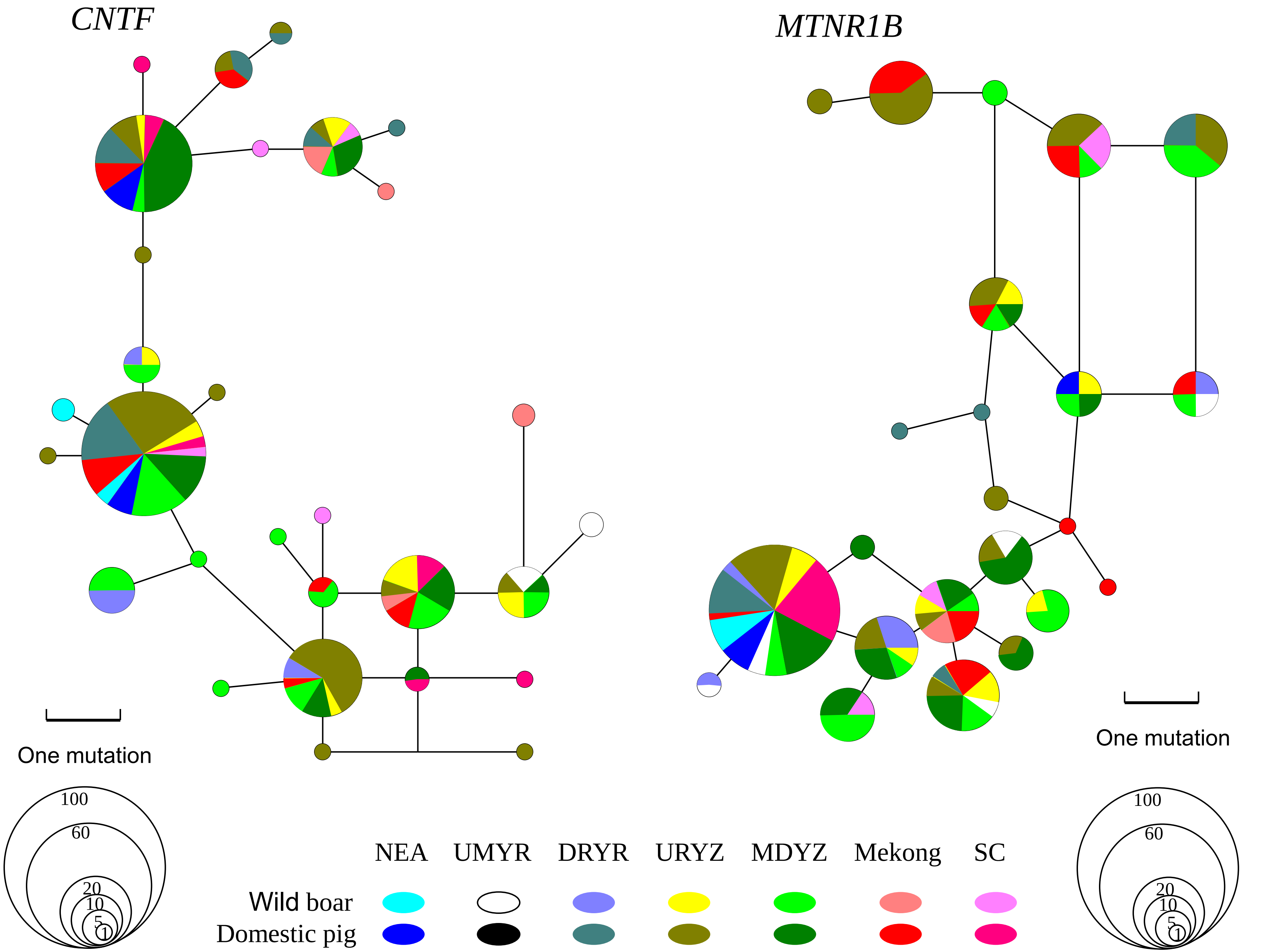

CNTF
MTNR1B
One mutation
One mutation
100
60
20
10
5
1
100
60
20
10
5
1
NEA
UMYR
DRYR
URYZ
MDYZ
Mekong
SC
Wild boar
Domestic pig

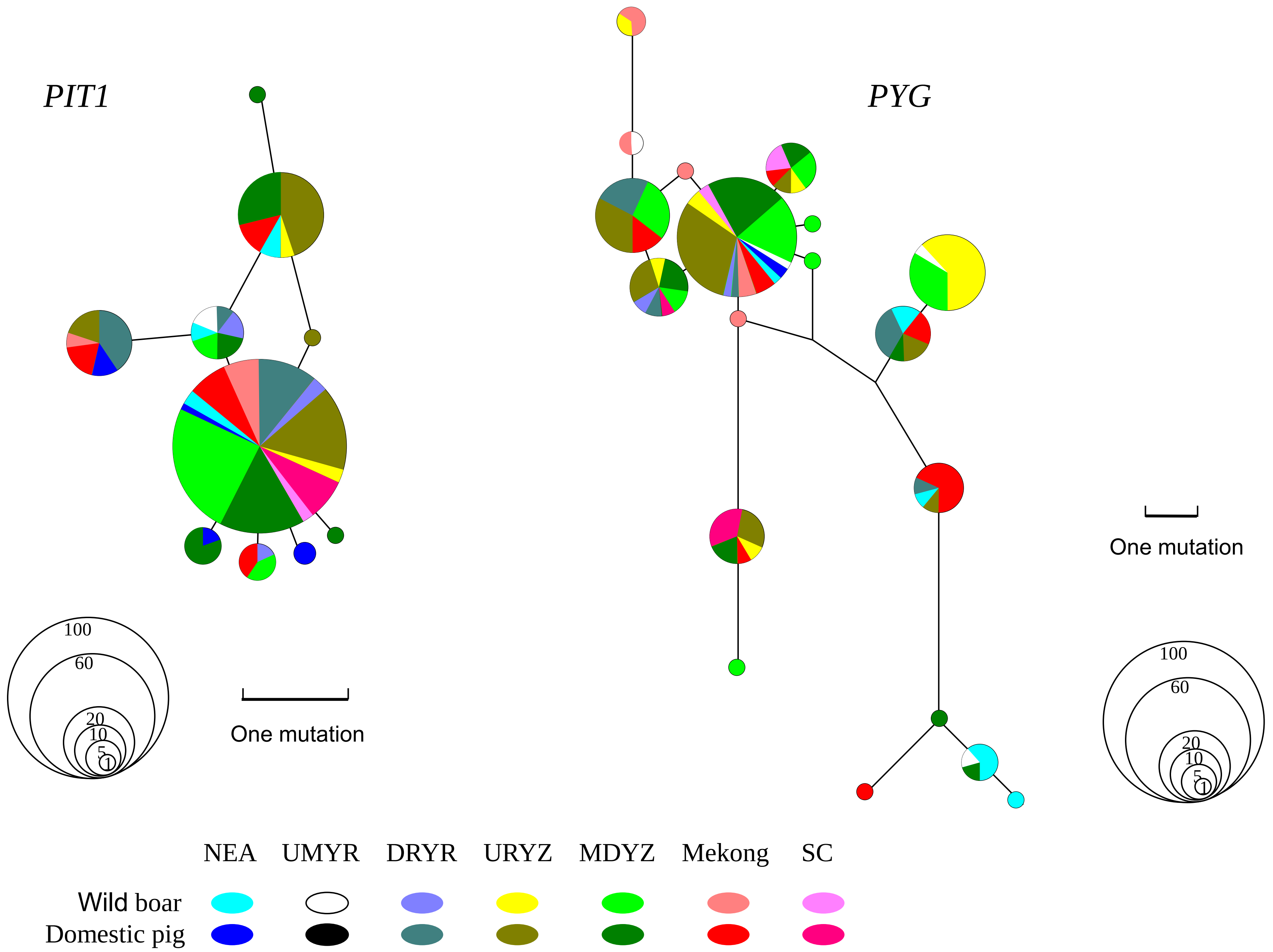
PIT1
PYG
100
60
20
10
5
1
One mutation
One mutation
100
60
20
10
5
1
NEA
UMYR
DRYR
URYZ
MDYZ
Mekong
SC
Wild boar
Domestic pig

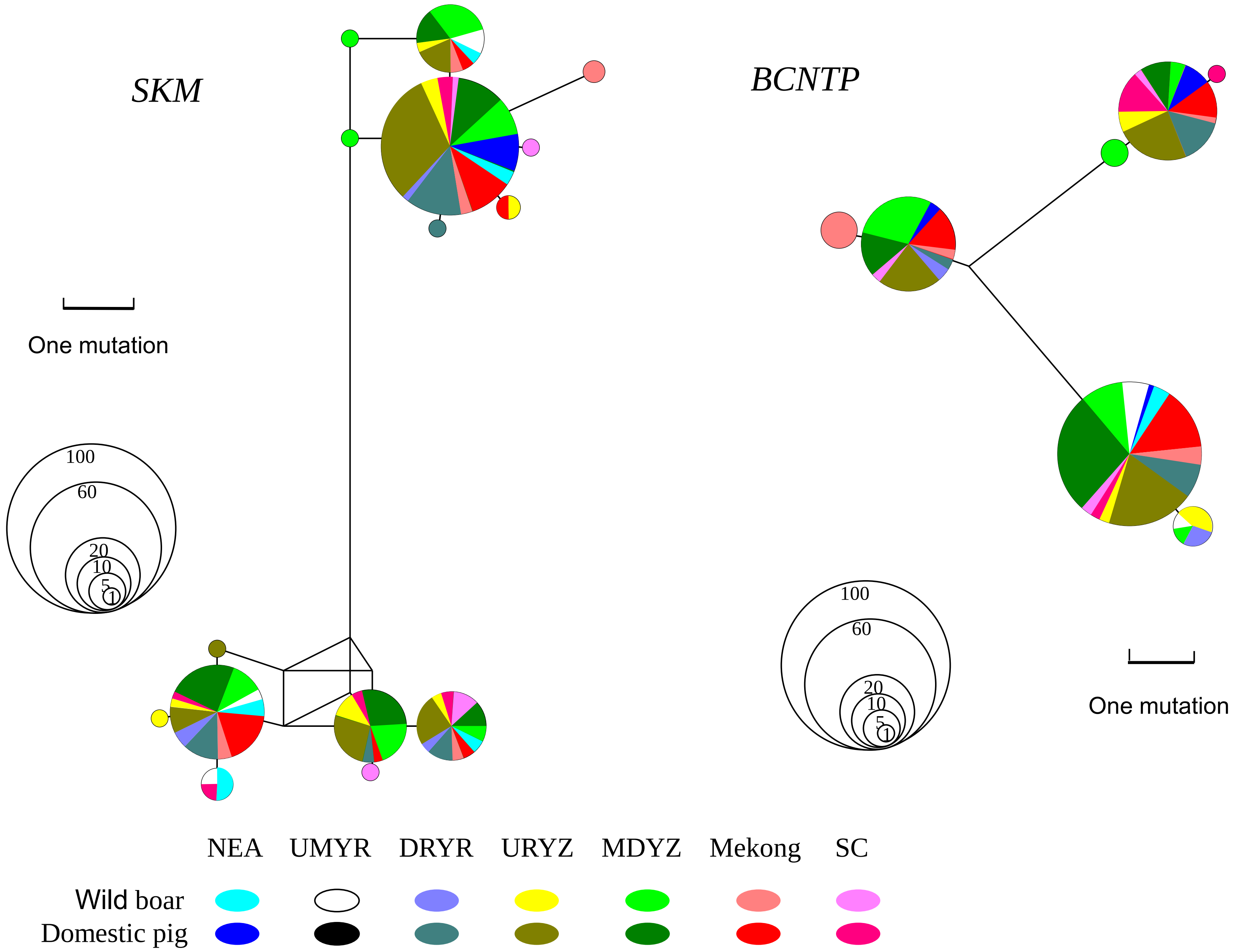
SKM
BCNTP
One mutation
100
60
20
10
5
1
100
60
20
10
5
1
One mutation
NEA
UMYR
DRYR
URYZ
MDYZ
Mekong
SC
Wild boar
Domestic pig

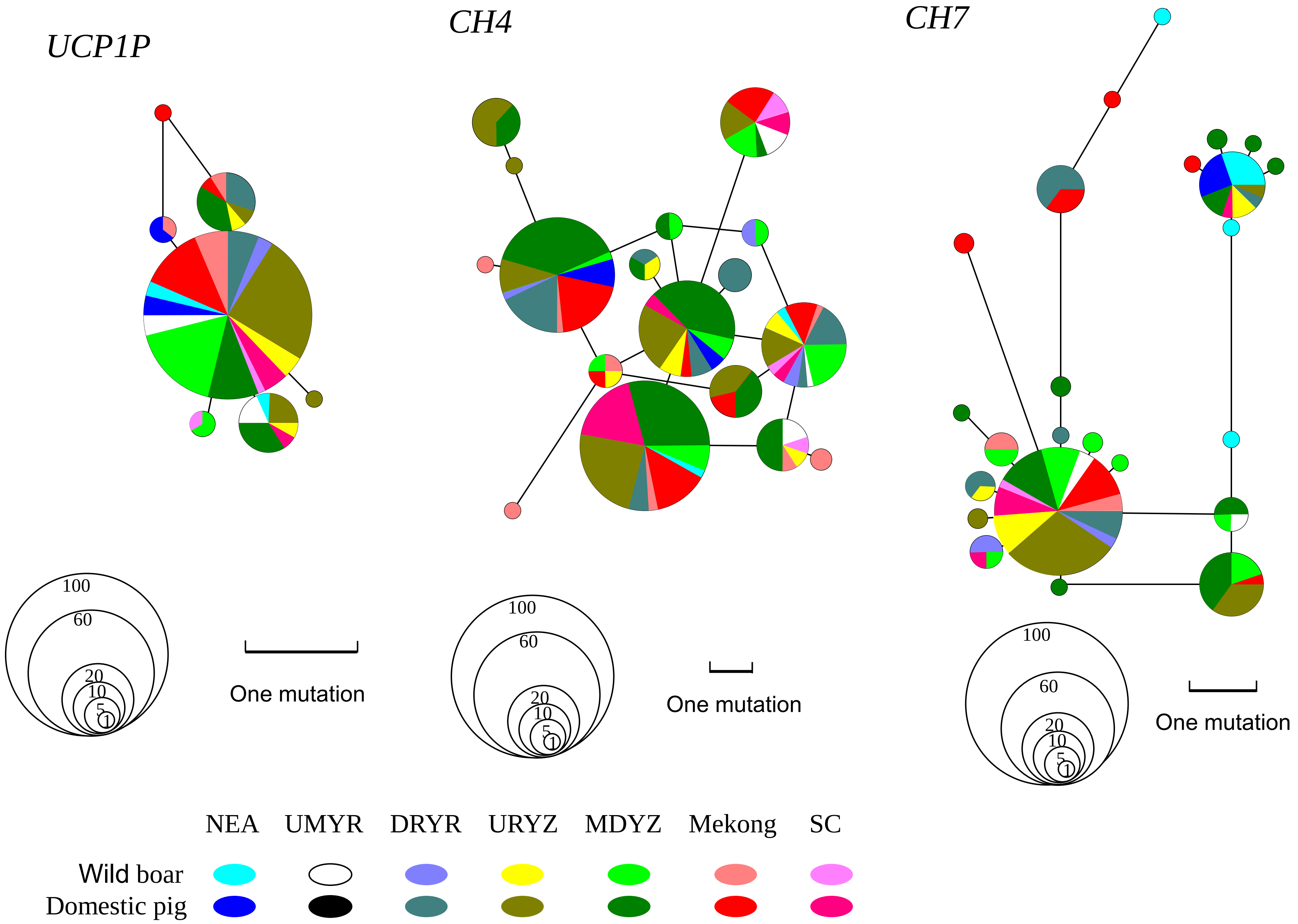

UCP1P
CH4
CH7
100
60
20
10
5
1
One mutation
NEA
UMYR
DRYR
URYZ
MDYZ
Mekong
SC
Wild boar
Domestic pig

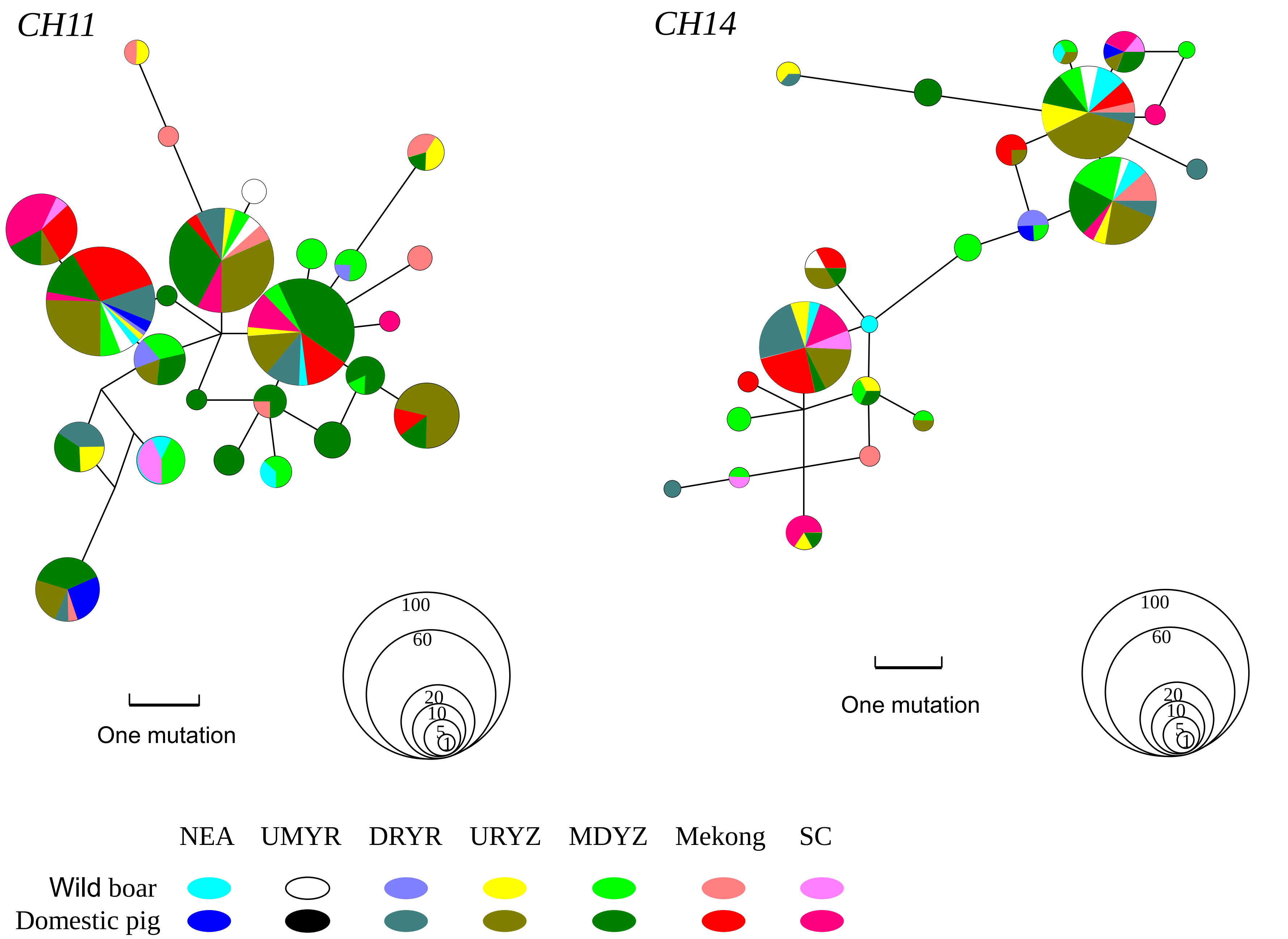
CH11
CH14
One mutation
One mutation
100
60
20
10
5
1
NEA
UMYR
DRYR
URYZ
MDYZ
Mekong
SC
Wild boar
Domestic pig

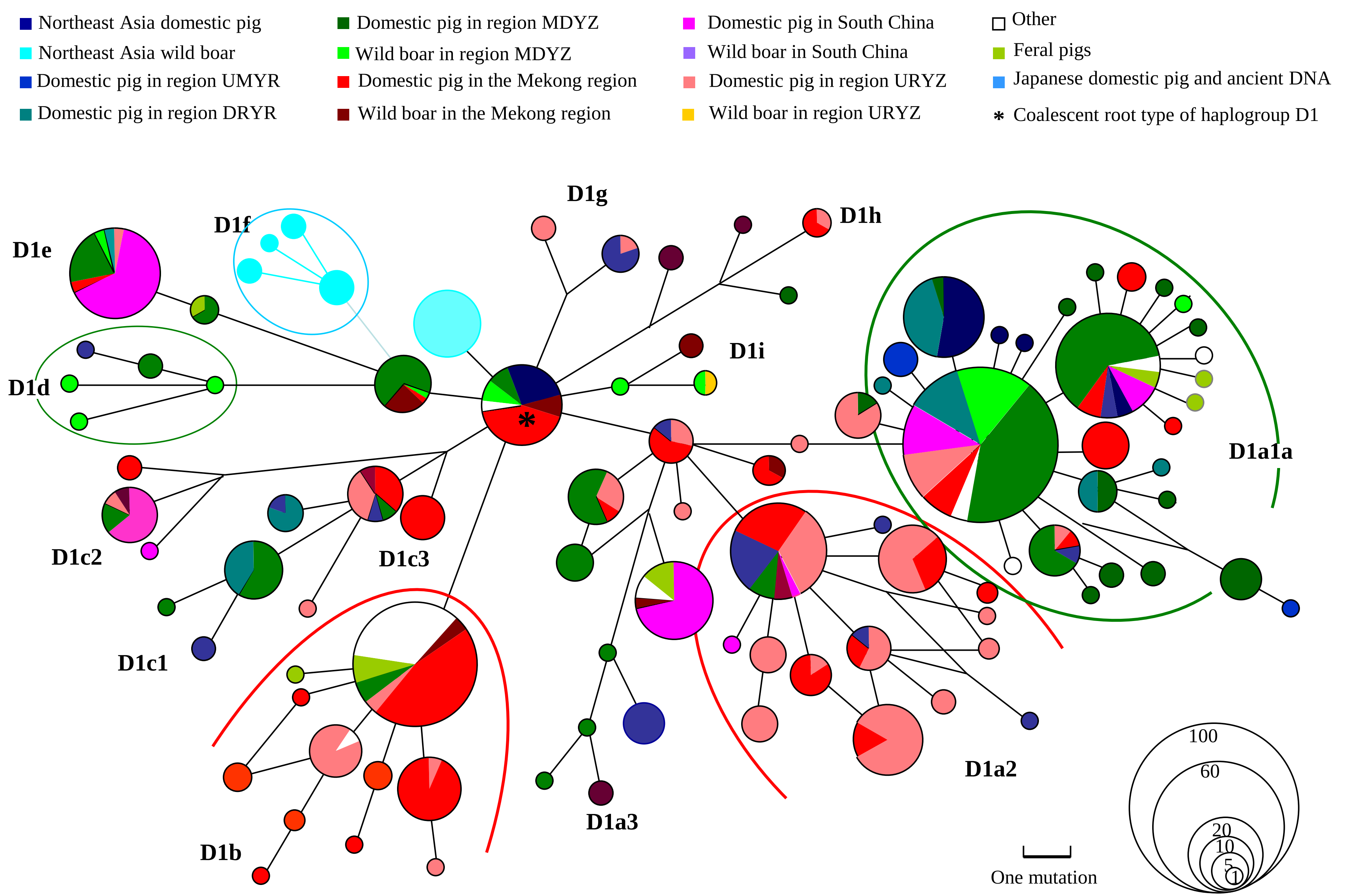

Northeast Asia domestic pig
Northeast Asia wild boar
Domestic pig in region UMYR
Domestic pig in region DRYR
Domestic pig in region MDYZ
Wild boar in region MDYZ
Domestic pig in the Mekong region
Wild boar in the Mekong region
Domestic pig in South China
Wild boar in South China
Domestic pig in region URYZ
Wild boar in region URYZ
Other
Feral pigs
Japanese domestic pig and ancient DNA
* Coalescent root type of haplogroup D1
D1e
D1f
D1g
D1h
D1i
D1d
D1a1a
D1c2
D1c3
D1c1
D1a2
D1a3
D1b
One mutation
100
60
20
10
5
1
